# Facile Construction of Flame-Resistant and Thermal-Insulating Sodium Alginate Aerogel Incorporating N- and P-Elements

**DOI:** 10.3390/polym16192814

**Published:** 2024-10-04

**Authors:** Ju Liu, Huanhui Zhan, Jianan Song, Chenfei Wang, Tong Zhao, Bo Fu

**Affiliations:** 1 Nantong Institute of Technology, College of Safety Engineering and Emergency Management, Nantong 226002, China; liuju@njfu.edu.cn; 2 Jiangsu Co-Innovation Center of Efficient Processing and Utilization of Forest Resources, College of Chemical Engineering, Nanjing Forestry University, Nanjing 210037, China

**Keywords:** aerogel, sodium alginate, flame retardant, thermal insulation, synergistic effect

## Abstract

In this study, sodium alginate (SA) aerogel cross-linked with Ca^2+^ was selected as the basic skeleton to construct a lightweight, flame retardant, and thermal insulating composite aerogel via modification with melamine and phytic acid. The resulting aerogel, SA-1.0 MP, achieved a thermal conductivity as low as 0.0379 W/(m·K). Compared to pristine SA aerogel, SA-1.0 MP demonstrated improved fire resistance, evidenced by a substantial increase in the limiting oxygen index (LOI) from 21.5% to 48.8% and a V-0 rating in the UL-94 test. Furthermore, a synergistic mechanism was proposed to explain its remarkable flame-retardant capability.

## 1. Introduction

Influenced by urbanization and global climate change, energy consumption in the building sector is increasing annually [1,2]. Developing high-performance building insulation materials can significantly reduce building energy consumption, offering a profound solution to the global energy crisis [3,4]. Aerogel, a solid material with a three-dimensional (3D) porous structure, is highly effective in thermal insulation owing to its low density, high porosity, and low thermal conductivity, making it a cutting-edge candidate for building insulation [5,6]. With the emphasis on sustainable development and carbon neutrality, increasing efforts are being attempted in the development and promotion of bio-based aerogels [7,8,9,10]. In comparison with petroleum-derived aerogels, bio-based aerogels exhibit outstanding characteristics, such as environmentally friendliness, biocompatibility, biodegradability, and renewability [11,12,13,14].

Among various natural polysaccharides, sodium alginate (SA), isolated from seaweed, has been extensively studied as a raw material for aerogel preparation due to its easy gelling [15,16]. The molecular structure of SA consists of β-D-mannuronic acid (M) and α-L-guluronic acid (G). The G blocks can chelate with multi-valence metal ions to form a stable “egg-box” structure [17]. This semi-rigid network gives sodium alginate-based aerogel intrinsic mechanical robustness [18]. In addition, the incorporation of multi-valence metal cations is conducive to the char-forming reaction of natural polymers, contributing to lower flammability. To date, the combination of diverse metallic ions with alginate for the enhancement of flame retardancy has attracted widespread attention [19,20]. However, pure SA aerogel, dominated by C, H, and O elements, is still highly susceptible to combustion and, thus, cannot meet strict safety requirements. Therefore, it is necessary to significantly improve the flame retardancy of pure SA aerogels without compromising their thermal insulation properties.

With the growing demand for green and non-toxic materials, flame retardants containing phosphorus and nitrogen have increasingly replaced halogen-based flame retardants [21,22]. As a representative phosphorus-based flame retardant, biomass-derived phytic acid (PA) is favored in flame-retardant applications due to its sustainable sourcing, low cost, and bio-affinity [23]. For example, Wang et al. impregnated PA into wood to produce a flame-retardant wood-based building material [24]. Nonetheless, natural polymers treated with PA alone show relatively low flame-retardant efficiency. To enhance flame retardancy, the nitrogen–phosphorus (N–P) coexisting flame-retardant system is commonly adopted [25,26,27]. For instance, Liu et al. fabricated a flame-retardant cotton fabric through the interfacial layer-by-layer assembly of phytic acid and urea [28]. Ding et al. obtained composite cellulose foams with competitive flame retardancy by integrating phytic acid and guanazole [29]. Cone calorimeter tests showed that the peak of the heat release rate and total heat release were 49.4% and 42.4% lower than those of pure cellulose foam, respectively. Due to its nitrogen-rich nature, organic base melamine (MEL) can act in synergism with P-containing compounds [30]. Specifically, the presence of abundant amino groups in melamine offers the potential for effective interaction with phytic acid (PA).

In this study, we present a facile approach to obtain a thermal insulating sodium alginate aerogel carrying a complex of phytic acid (PA) and melamine (MEL). Based on the inherent abundant functional groups, a strong and stable integration of flame retardant with aerogel matrix was achieved via the supramolecular self-assembly strategy, greatly improving the flame retardance of SA aerogel. The high porosity of the composite aerogels results in low thermal conductivity, making them suitable for heat insulation. Experimental results also demonstrated that the composite aerogels have integrated water stability and high mechanical properties. This study provides a new perspective on the functionalization of sodium alginate aerogel.

## 2. Experimental

### 2.1. Materials

Sodium alginate (S100128, with a M/G ratio of 1:1) [31], phytic acid (PA, 70%), and melamine (MEL, 99%) were obtained from Shanghai Aladdin Biochemical Technology Co., Ltd., Shanghai, China. Anhydrous calcium chloride (CaCl_2_) was supplied by Sinopharm Chemical Reagent Co., Ltd. (Beijing, China). Deionized (DI) water used throughout the experiment was prepared using a laboratory water purification system.

### 2.2. Preparation of SA-MP Composite Aerogel

Firstly, a specific amount of sodium alginate powder was dispersed into DI with magnetic stirring to prepare a 1.6 wt% SA solution. After removing air bubbles, the homogeneous SA solution was poured into molds and frozen entirely in liquid nitrogen. Pure SA aerogels were produced by drying in a vacuum freeze-dryer at −60 °C for 12 h. The obtained pure SA aerogels were then immersed in a 1 wt% CaCl_2_ solution. Following that, the aerogels were soaked in deionized water for 24 h to remove excess Ca^2+^ ions. The SA aerogels crosslinked with Ca^2+^ (referred to as SA-Bare) were obtained after a secondary lyophilization process and subsequently subjected to supramolecular self-assembly using MEL-PA (MEL to PA molar ratio of 6:1). Taking SA-1.0 MP as an example: a certain amount of MEL was completely dissolved in DI at a controlled temperature of 80 °C. Subsequently, SA-Bare aerogels were added to the above MEL solution and allowed to react for 15 min. Afterward, PA solution was added dropwise to the reaction mixture, and the reaction was held for another 15 min. Finally, the aerogels were soaked in deionized water for 24 h to remove excess reaction products and again freeze-dried to prepare the final samples. Based on the initial MEL-PA concentrations (0.2 wt%, 0.6 wt%, and 1.0 wt%), the aerogels were designated as SA-0.2 MP, SA-0.6 MP, and SA-1.0 MP, respectively.

### 2.3. Characterizations

Chemical structures were characterized using an FTIR spectrometer (Brucker VERTEX 80 V, Billerica, MA, USA) in attenuated total reflection mode over a range of 400–4000 cm^−1^. The microstructure and the elemental dispersion of samples were examined using a Regulus 8100 scanning electron microscope (SEM) (Hitachi, Tokyo, Japan). Compressive properties were assessed at 80% strain using a universal testing machine (CMT850) (Jinan Liangong Testing Technology Co., Ltd., Jinan, China) at a compression speed of 10 mm/min.

The limiting oxygen index (LOI) test was conducted using a JF-3 oxygen index measuring instrument (Beijing Zongheng Shidai Technology Co., Ltd., Beijing, China). The vertical burning test (UL-94) was performed in accordance with the GB/T 8333-2008 standard. Targeted samples used for the combustion tests were 100 mm × 10 mm × 10 mm in tangential, radial, and longitudinal directions, respectively. Each sample was tested in triplicate. Cone calorimetry testing (CCT) was performed on a cone calorimeter (WK5243-PC, TESTech (Suzhou) Instrument Technologies Co., Ltd., Suzhou, China) at a heat flux of 35 kW/m^2^ based on the ISO5660 standard. Thermal conductivity was measured using a thermal conductivity meter (TC3000E, Xi’an Xiatech Electronic Technology Co., Ltd., Xi’an, China) at 25 °C. Thermal imaging maps of samples were recorded using a thermal infrared imager (T1050sc, FLIR, Wilsonville, OR, USA). Thermal decomposition of the specimens was studied using a thermogravimetric analyzer (NETZSCH TG 209F, Selb, germany) under a N_2_ atmosphere with a flow rate of 20 mL·min^−1^, from 30 to 800 °C at a heating rate of 10 °C·min^−1^. The leftover char residues were analyzed using a DXR532 laser Raman spectrometer (Thermo Fisher Scientific, Waltham, MA, USA).

## 3. Results and Discussion

### 3.1. Material Characterizations

**Figure 1:**
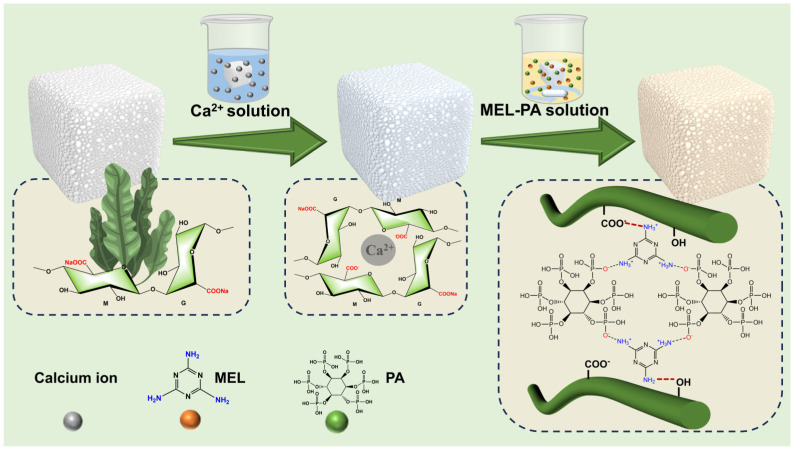
Schematic diagram of the preparation process of SA-MP composite aerogels.

As shown in Figure 1, the hybrid aerogel with fire-retardant properties was fabricated using an in situ supramolecular self-assembly strategy, in which MEL and PA were sequentially introduced onto the cell walls of SA aerogel. Prior to assembly, SA aerogel was prepared applying an ice-crystal templating method and then immersed in a Ca^2+^ solution, resulting in a stable porous structure induced by Ca^2+^ cations [32]. This Ca^2+^-mediated coordination mode mainly occurs at the G residues in sodium alginate [33,34]. MEL-containing amine groups can be easily adsorbed onto the SA aerogel rich in carboxyl and hydroxyl groups through electrostatic and hydrogen bonding interactions [35]. As PA was gradually added, the amine groups of MEL on the aerogel cell walls served as active sites for interaction with the anionic phosphate groups [36]. This assembly method, which relies on group linkage, facilitates the attachment of flame retardants to the matrix.

**Figure 2:**
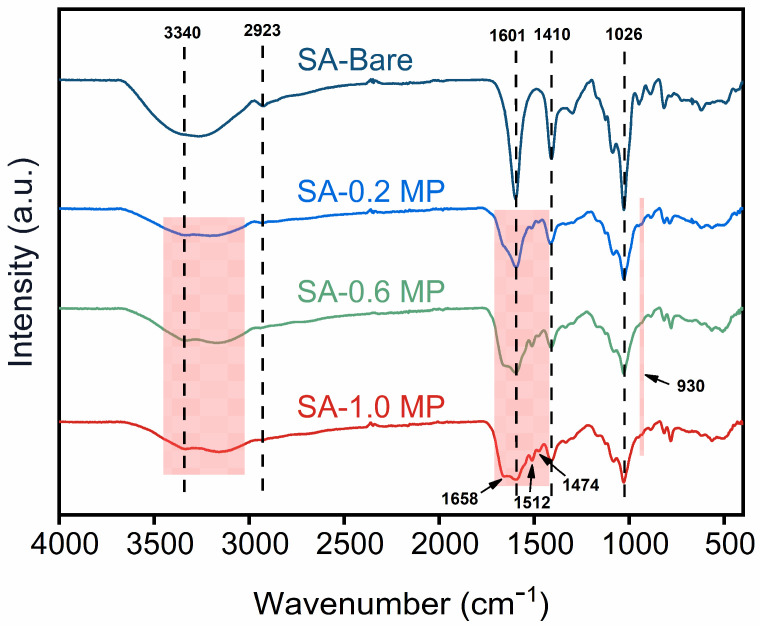
FT-IR spectra of SA-Bare, SA-0.2 MP, SA-0.6 MP, and SA-1.0 MP.

Figure 2 presents the results of FTIR employed to investigate the functional groups of aerogels. The broad absorption band around 3340 cm^−1^ and the shoulder peak at 2923 cm^−1^ were allocated, respectively, to stretching vibration of –OH and C–H bonds. The primary peaks at 1601 cm^−1^ and 1410 cm^−1^ corresponded to the asymmetric and symmetric stretching vibrations of carboxylate salt groups [37]. The strong absorption peak observed at 1026 cm^−1^ was assigned to the skeletal vibration of C–O–C glycoside bonds in the pyranose ring. In addition to the characteristic peaks of SA, the flame-retardant modified specimens also exhibited spectral features associated with PA and MEL. The new peak at 930 cm^−1^ might originate from the stretching vibration of P–O [38]. Characteristic peaks emerging at 1658 cm^−1^, 1512 cm^−1^, and 1474 cm^−1^ were typical of the triazine ring in MEL [39]. However, some subtle distinctions were found following the introduction of flame retardants. Compared to SA-Bare, the –OH stretching vibrational peak of composite aerogels appeared widened and weakened, which was caused by the overlap with the –NH_2_ stretching vibration from melamine and the formation of hydrogen bonds between melamine and sodium alginate [40]. Additionally, the reduced peak intensity of carboxyl groups indicated that the carboxylate of polysaccharide chains was partially consumed by the amino groups of MEL [41]. The above analysis confirmed the successful surface modification of SA aerogel through the supramolecular assembly of MEL-PA.

**Figure 3:**
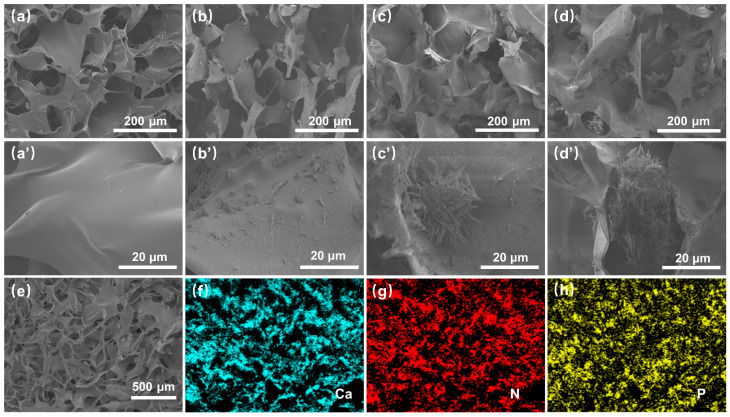
SEM images of SA-Bare (**a**,**a′**), SA-0.2 MP (**b**,**b′**), SA-0.6 MP (**c**,**c′**), SA-1.0 MP (**d**,**d′**), and EDS mapping images of SA-1.0 MP (**e**–**h**).

The internal microstructure and morphology of the aerogel were observed by SEM (Figure 3). Pure SA aerogel exhibited a highly interconnected three-dimensional porous structure (Figure 3a). All the composite aerogels modified with MEL-PA presented an internal pore structure similar to that of SA-Bare (Figure 3b–d). High-resolution images clearly showed that the cell wall surface of pure SA aerogel was smooth (Figure 3a′). In contrast, nanorod aggregates of micron size were found on the surface of the composite aerogel, and the eventual size of nanorod aggregates increased with the initial MEL-PA concentration (Figure 3b′–d′), demonstrating that the MEL-PA assembled structure was successfully attached to the aerogel. Furthermore, the element mapping images revealed that N and P elements were homogeneously distributed on the SA-1.0 MP aerogel (Figure 3g,h), suggesting uniform coverage of phytic acid and melamine on the flammable substrate.

As shown in Figure S1, the macro-size determined using a digital caliper revealed that pure SA aerogel exhibited observable shrinkage after cross-linking with Ca^2+^. The length of the square sample decreased from the original 95.05 mm to 83.93 mm, resulting in a decline in size of 11.7%. Water stability testing showed that pure SA aerogel consists of hydrophilic groups quickly collapsed and completely dissolved in water. Conversely, the Ca^2+^ cross-linked aerogel maintained its structural integrity even after being immersed in deionized water for 48 h, demonstrating its robust structure and excellent water stability. This structural stability is essential for the subsequent supramolecular assembly process to create stable ternary composite aerogels.

**Figure 4:**
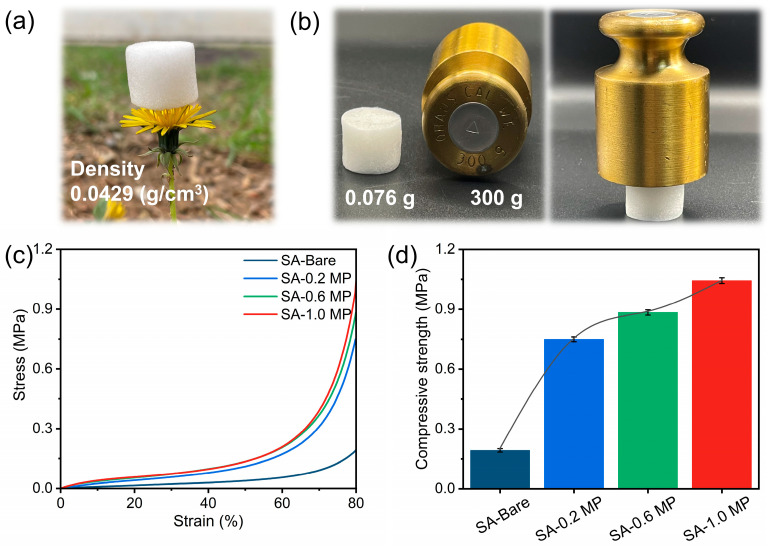
(**a**) Digital photo of SA-1.0 MP standing on top of a flower, (**b**) digital photograph of SA-1.0 MP subjecting weight, (**c**) compressive stress–strain curves, and (**d**) compressive strength with a strain of 80%.

It was observed that the SA-1.0 MP aerogel with an ultra-light density of 0.0262 g/cm^3^ could freely stand upright on top of a flower. Moreover, the excellent structural stiffness of the SA-1.0 MP aerogel enabled it to withstand approximately 4000 times its own weight without deformation. Compression experiments with 80% strain were performed on the aerogels to further evaluate their mechanical properties. As shown in Figure 4c, the stress–strain curves display three distinct regions: linear elastic (0–10% strain), plastic yield (10–60% strain), and densification (60–80% strain) [42]. Compared to the bare SA aerogel, the compressive strength of the composite aerogels increased with higher MEL-PA content, especially the compressive strength of SA-1.0 MP, which was 4.3 times greater than that of SA-Bare, reaching a maximum of 1.043 MPa. This improvement can be attributed to the increased density and reinforced cell walls resulting from interaction between the SA matrix and the MEL-PA supramolecular structure, which collectively strengthens the aerogel framework.

### 3.2. Fire-Retardant and Thermal Insulation Properties

**Figure 5:**
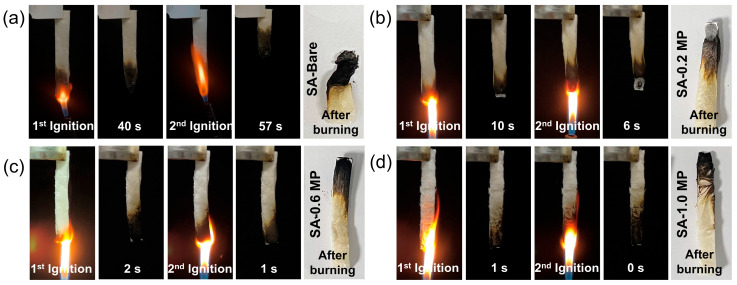
Vertical burning test results of SA-Bare (**a**), SA-0.2 MP (**b**), SA-0.6 MP (**c**), and SA-1.0 MP (**d**).

The digital photographs from the vertical combustion tests clearly illustrated the flammable behavior of the aerogels (Figure 5). When exposed to a flame, the SA-Bare aerogel burned rapidly, accompanied by a prolonged smoldering period, thus failing to meet UL-94 test requirements (Figure 5a). On the contrary, all the modified aerogels demonstrated a self-extinguishing performance after the igniter was removed (Figure 5b–d). For samples SA-0.6 MP and SA-1.0 MP, the total combustion time did not exceed 2 s in both the first ignition and the second ignition, comfortably passing the UL-94V-0 rating when compared with the standards. Compared to the unmodified SA aerogel, which nearly completely burned out during the vertical combustion experiment, the composite aerogels retained their shape and had significantly longer residual lengths. The LOI values, which indicate the fire resistance behavior of the aerogels, are summarized in Table 1. The LOI value of SA-Bare aerogel was 21.5%, while the composite aerogels reached substantially higher LOI values, which corresponded to the flame-retardant materials (LOI > 27%). These results indicated that the presence of MEL-PA significantly enhanced the flame retardancy of aerogels.

**Figure 6:**
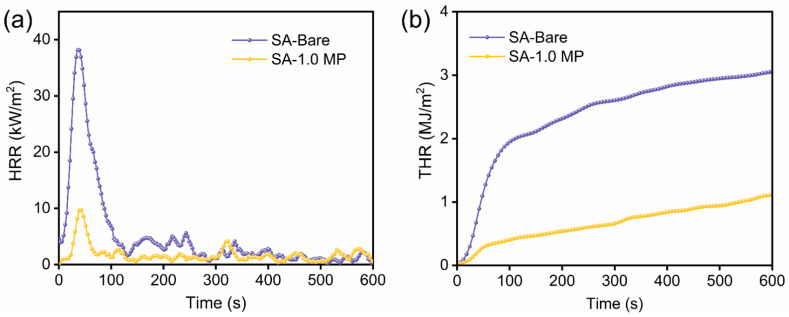
Heat release rate (**a**) and total heat release (**b**) curves of SA-Bare and SA-1.0 MP.

The combustion behavior of aerogels was further judged using a Cone calorimetry test (CCT), which can simulate a flame scene similar to real combustion. Relevant test data such as time to ignition (TTI), total heat release (THR), peak of heat release rate (PHRR), time to peak heat release rate (TpHRR), and fire growth index (FIGRA) are presented in Table 2. The heat release rate (HRR) and total heat release rate (THR) curves of SA-Bare and SA-1.0 MP aerogels are illustrated in Figure 6. Due to its rich hydrocarbon content, SA-Bare aerogel was easily ignited with a TTI of only 9 s and reached the maximum heat release rate of 38.5 kW/m^2^ at 37 s. After combining with MEL-PA, the composite aerogel became noncombustible. The time taken to reach PHRR was delayed to 41 s, and the corresponding PHRR value was reduced to 9.91 kW/m^2^, reflecting that combustion intensity was suppressed. Meanwhile, the total heat release THR value of SA-1.0 MP declined from 3.07 MJ/m^2^ for neat SA aerogel to 1.12 MJ/m^2^, resulting in a 63.5% reduction. It can be inferred that a dense carbon layer rapidly formed in the composite aerogel after ignition, isolating heat and oxygen, preventing further combustion, thereby reducing the PHRR and THR. In addition, as the fire growth index (FIGRA) can effectively estimate the fire safety of the material, the lower the FIGRA value, the higher its level of fire safety [43]. As shown in Table 2, the FIGRA of the SA-1.0 MP aerogel was 0.242 kW/(m^2^·s), 76.8% lower than that of SA-Bare aerogel, indicating a reduced fire risk. In summary, the inclusion of MEL and PA can improve the flame-retardant efficiency of SA aerogel and play a positive role in reducing fire hazards.

**Figure 7:**
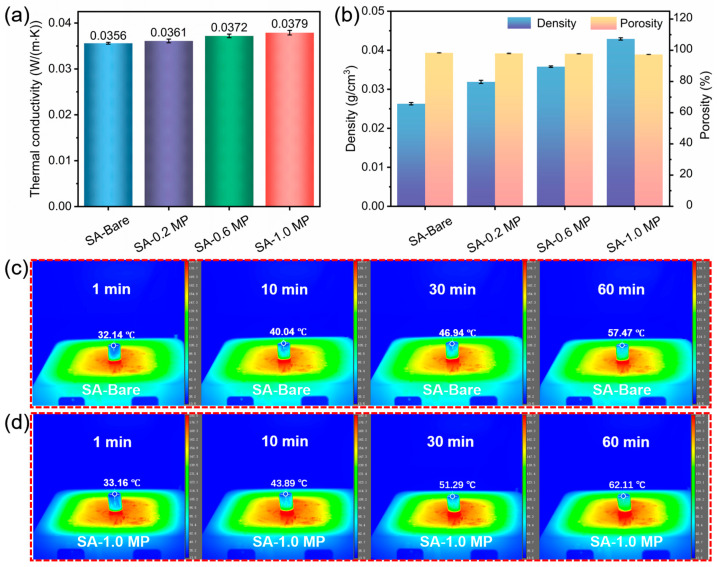
(**a**) Thermal conductivity of different samples, (**b**) density and porosity of different samples, (**c**) infrared thermal imaging of SA-Bare at a height of 2 cm on a hot stage at 200 °C, and (**d**) infrared thermal imaging of SA-1.0 MP at a height of 2 cm on a hot stage at 200 °C.

Thermal conductivity is the quantitative parameter for assessing thermal insulation performance. Table S1 provides detailed parameters, including thermal conductivity, density, and porosity of various aerogels. It should be noted that the thermal conductivity of the samples ranged from 0.03 W/(m·K) to 0.04 W/(m·K), highlighting effective thermal insulation performance. However, the introduction of MEL-PA into the SA matrix resulted in an increase in overall thermal conductivity, with the increment positively correlated to the concentration of MEL-PA (Figure 7a). Despite the increased density of the modified aerogel reducing porosity and causing a rise in thermal conductivity, the three-dimensional porous structure and high porosity still endow SA-based aerogels with satisfactory thermal insulation performance [44].

To further verify the heat transfer distribution and thermal insulation properties of the aerogels, the samples were placed on a heating platform at a constant temperature of 200 °C. The upper surface temperature was recorded using infrared thermography from 1 to 60 min (Figure 7c,d). The upper surface temperature of aerogels increased according to the extension of time, reaching 57.47 °C for SA-Bare and 62.11 °C for SA-1.0 MP after 60 min. SA-Bare exhibited superior thermal insulation capability, blocking 71.3% of heat. Due to the higher thermal conductivity value, SA-1.0 MP possessed a smaller amount of heat insulation (68.9%). Overall, the prepared aerogels demonstrated improved heat insulation performance at elevated temperatures compared to other reported SA-based aerogels [42,45]. The three-dimensional porous structure of aerogels not only extends the heat conduction path but also effectively blocks heat radiating upward from the heating platform [46].

### 3.3. Flame Retardant Mechanism

**Figure 8:**
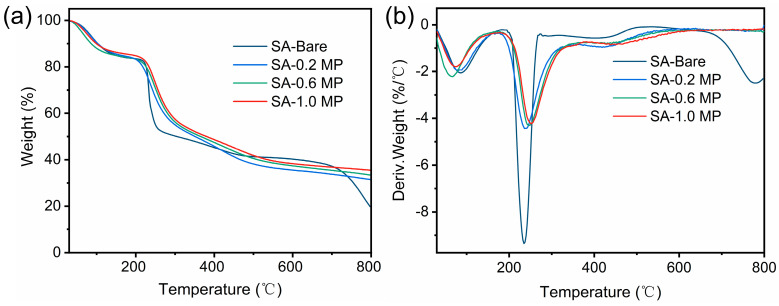
TG (**a**) and DTG curves (**b**) of SA-Bare, SA-0.2 MP, SA-0.6 MP, and SA-1.0 MP under N_2_ atmosphere.

The thermal stability of the aerogel in an N_2_ atmosphere was studied using thermogravimetric analysis (TGA). The TG and DTG curves are shown in Figure 8, with corresponding parameters listed in Table 3. A slight weight loss of around 100 °C was observed for all samples, which can be ascribed to the loss of moisture absorbed from the air. The main decomposition temperature of aerogels ranged from 180 to 400 °C. Within this temperature range, hydrogen bond cleavage, glycosidic bond rupture, and decarbonylation of the SA backbone occurred, resulting in the production of H_2_O and CO_2_ [18]. The temperature for the 10 wt% loss (T_d10%_) of SA-Bare was 102.4 °C while that of the composites all increased due to the presence of PA that can lower the initial thermal decomposition temperature of aerogels [45]. The temperature corresponding to the maximum mass loss rate (T_d max_) increased, and the maximum decomposition rate in the DTG curves decreased as a result of the introduction of MEL-PA. Furthermore, the final char yield of composite aerogels at 800 °C dramatically increased, with the highest residual weight percentage reaching 35.64% for SA-1.0 MP. These results confirmed the role of the flame retardant, fabricated through MEL-PA self-assembly, in inhibiting the pyrolysis of the aerogel skeleton and enhancing high-temperature stability.

**Figure 9:**
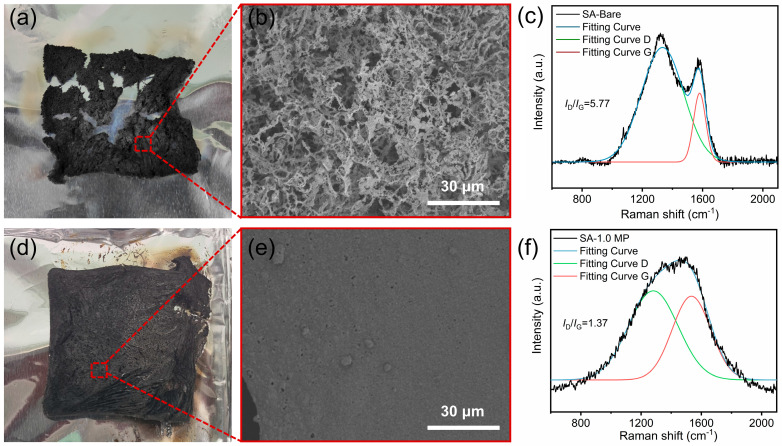
Digital photographs and SEM images of char residues of SA-Bare aerogel (**a**,**b**), and SA-1.0 MP aerogel (**d**,**e**). Raman fitting data of SA-Bare (**c**), and SA-1.0 MP (**f**).

Digital photos and SEM images of the aerogels after Cone calorimetry testing are shown in Figure 9. The SA-Bare skeleton shrank and broke during combustion, leaving minimal char residue with a loose and disordered structure. The non-compact char layer failed to prevent contact between the substrate and external oxygen, leading to the diffusion of flammable gases and heat transfer during combustion. In contrast, the SA-1.0 MP aerogel retained its original shape after combustion. As depicted in Figure 9d,e, the char layer exhibited significant integrity with multiple small pores on its surface. During the burning process of SA-1.0 MP aerogel, a high-quality char layer formed, accompanied by the release of non-flammable gases from MEL-PA thermal decomposition, synergistically enhancing the flame-retardant performance.

Raman spectroscopy is an effective method for characterizing the orderliness of residual char. The Raman spectra showed two prominent peaks at 1340 cm^−1^ and 1580 cm^−1^, corresponding to the D band (amorphous carbon) and G band (graphite structure), respectively. The graphitization degree can be calculated by the integrated intensity of D peak and G peak (I_D_/I_G_). A smaller I_D_/I_G_ value indicates a higher degree of graphitization, prompting a denser and more stable char layer [47]. It is encouraging to see that the I_D_/I_G_ value decreased from 5.77 for SA-Bare to 1.37 for SA-1.0 MP (Figure 9c,f). This result demonstrates that the introduction of MEL-PA promoted graphitization on the surface of substrates, which is consistent with the compact and continuous carbon structure found in the SEM images.

**Figure 10:**
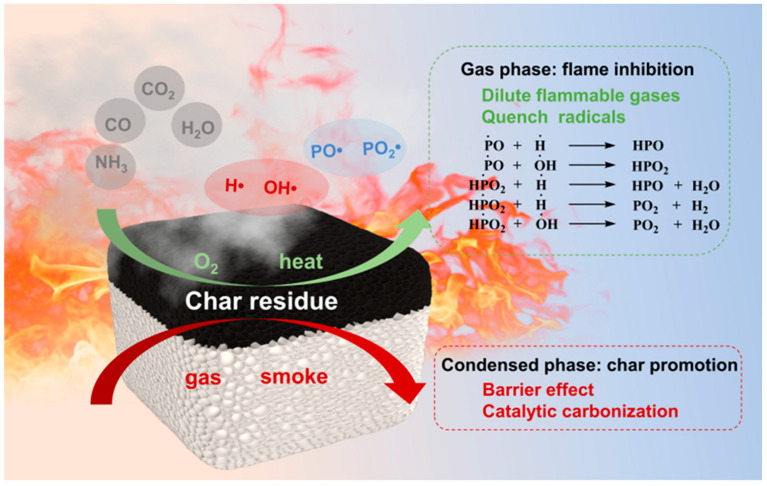
Schematic illustration of the flame retardancy mechanism of SA-1.0 MP aerogel during combustion.

Based on the above analysis, a potential flame-retardant mechanism for the SA-1.0 MP aerogel during combustion is produced in Figure 10. The flame-retardant mechanism generally involves a synergistic effect between the gas phase and the condensed phase. When the SA-1.0 MP aerogel encountered flame combustion, the decomposition of MEL released a large amount of NH_3_ [30], while the burning of the SA framework produced non-flammable gases (H_2_O and CO_2_), which can dilute the concentration of oxygen and combustible gas. Simultaneously, the phosphate groups in PA decompose at elevated temperatures, generating radicals such as PO·and PO_2_, which check active radicals such as H·and OH·in the combustion reaction, thereby terminating the chain propagation [48]. Additionally, phosphoric or polyphosphoric acids from PA pyrolysis at elevated temperatures are crucial for condensed-phase flame retardancy. These derivatives can serve as acid sources, promoting the dehydration of SA to form a compact and continuous carbon layer. The formed dense carbon layer acts as a physical barrier, effectively restricting the transfer of oxygen and heat, isolating the underlying framework from the flame, and protecting the substrate from further decomposition. Summarily, the incorporation of MEL-PA containing N and P elements synergistically renders the aerogel with greater flame retardancy.

## 4. Conclusions

To meet the demand for energy-efficient and safe thermal insulation materials, this study developed an aerogel derived from sodium alginate. Benefiting from the abundant hydroxyl and carboxyl groups of the SA, the in situ supramolecular assembly of melamine and phytic acid onto the aerogel surface was successfully achieved, imparting inherent flame retardancy to the resultant aerogel. The composite aerogel SA-1.0 MP presented effective fire safety properties, reaching a high LOI value of 48.8%, passing the UL 94 V-0 rating. Furthermore, the highly porous aerogel possessed low thermal conductivity (0.0379 W/(m·K)) and demonstrated outstanding thermal insulation capability at 200 °C. This study offers a potential strategy for designing flame-retardant and heat-insulating bio-based aerogels.

## Figures and Tables

**Table 1:** Limiting oxygen index and UL-94 test results of different aerogels.

Sample	LOI (%)	Dripping	t_1_/t_2_ (s)	UL-94
SA-Bare	21.5	No	40/57	NR
SA-0.2 MP	28.3	No	10/6	V-0
SA-0.6 MP	37.2	No	2/1	V-0
SA-1.0 MP	48.8	No	1/0	V-0

**Table 2:** CCT data of SA-Bare and SA-1.0 MP.

Sample	TTI (s)	THR (MJ/m^2^)	PHRR (kW/m^2^)	TpHRR (s)	FIGRA (kW/(m^2^·s))
SA-Bare	9	3.07	38.5	37	1.041
SA-1.0 MP	-	1.12	9.91	41	0.242

**Table 3:** Thermogravimetric parameters of different aerogels.

Sample	T_d10%_ (°C)	T_d max_ (°C)	C_y800_ (%)
SA-Bare	102.4	232.6	19.83
SA-0.2 MP	84.8	238.6	31.58
SA-0.6 MP	96.9	247.6	33.43
SA-1.0 MP	100.4	253.5	35.64

## Data Availability

The data will be available upon reasonable request.

## Supplementary Materials

The following supporting information can be downloaded at: https://www.mdpi.com/article/10.3390/polym16192814/s1, Figure S1: Digital photos of size comparison of pure SA aerogel before and after Ca^2+^ cross-linking (a); digital photos of water stability test of pure SA aerogel before and after Ca^2+^ cross-linking (b); Table S1: Density, porosity and thermal conductivity of different aerogels.
