# Supplementary material for: Facile Construction of Flame-Resistant and Thermal-Insulating Sodium Alginate Aerogel Incorporating N- and P-Elements
Source: Polymers (Basel). 2024 Oct 4;16(19):2814. doi: 10.3390/polym16192814 (PMC11479020; doi:10.3390/polym16192814)
Supplement: Supplementary file 1 [file polymers-16-02814-s001.zip › polymers-3230838-supplementary.pdf]

Supporting information

for

Facile construction of Flame-Resistant and Thermal-Insulating Sodium alginate Aerogel incorporating N-and P-elements

Ju Liu[a,b], Huanhui Zhan[b], Jianan Song[b], ChenFei Wang[b], Tong Zhao[b], Bo Fu[b,*]

[a] College of Safety Engineering and Emergency Management, Nantong Institute of Technology, Nantong 226002, China.

[b] Jiangsu Co-Innovation Center of Efficient Processing and Utilization of Forest Resources, College of Chemical Engineering, Nanjing Forestry University, Nanjing 210037, China.

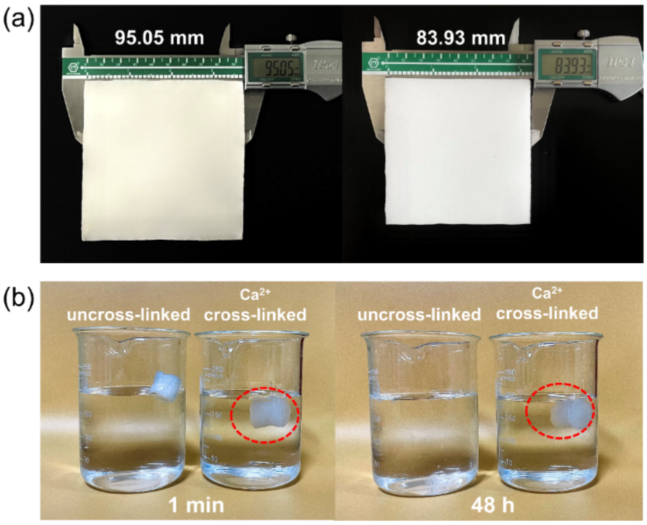

Figure S1. Digital photos of size comparison of pure SA aerogel before and after Ca^{2+} cross-linking (a); digital photos of water stability test of pure SA aerogel before and after Ca^{2+} cross-linking (b)

Table S1. Density, porosity and thermal conductivity of different aerogels

Sample	Density (g/cm^3)	Porosity (%)	Thermal conductivity (W/(m·K))
SA-Bare	0.0263	98.35	0.0356
SA-0.2 MP	0.0319	97.99	0.0361
SA-0.6 MP	0.0358	97.75	0.0372
SA-1.0 MP	0.0429	97.30	0.0379
